# Treatment of Postsurgical Scalp Scar Deformity Using Follicular Unit Hair Transplantation

**DOI:** 10.1155/2019/3423657

**Published:** 2019-05-13

**Authors:** Hyokyung Yoo, Jaeseong Moh, Ji-Ung Park

**Affiliations:** ^1^Department of Plastic and Reconstructive Surgery, Seoul National University Boramae Hospital, Seoul National University College of Medicine, Seoul, Republic of Korea; ^2^Dr. Moh Hair Transplantation Center, Seoul, Republic of Korea

## Abstract

**Background:**

Although stable cicatricial alopecia (SCA) secondary to surgical events of the scalp can affect patients' psychosocial status, hair transplantation onto postsurgical scar tissue on the scalp is challenging because of tissue stiffness and poor blood circulation.

**Objective:**

In contrast to traditional surgical treatments, such as excision, local flap, and tissue expansion, follicular unit (FU) hair transplantation offers aesthetically pleasing results in the treatment of postsurgically induced SCA.

**Materials and Methods:**

This study included 15 patients with SCA of the scalp due to postsurgical scarring. The patients underwent a single session of hair transplantation of approximately 35 units/cm^2^ density. The graft survival rate was evaluated 12 months after the procedure. The Patient and Observer Scar Assessment Scale (POSAS) was used to analyze the preoperative and postoperative satisfaction.

**Results:**

The FUE hair transplantation had a mean survival rate of 80.67% (range 70-90%). The patient and observer satisfaction significantly improved after the procedure; the POSAS scores were 24.47 (range 16-38) preoperatively and 11.60 (range 7-18) postoperatively.

**Conclusion:**

FU hair transplantation could be an effective method for managing scar tissue on the scalp and offers several advantages, including a high transplantation survival rate and satisfactory postoperative results.

## 1. Introduction

Because hair plays an important role in an individual's social and psychological health, maintaining and preventing hair loss are major concerns of individuals of various ages and genders. Due to growing interest in and demand for cosmetic procedures, range has been broadened from male pattern baldness to different types of alopecia, such as hairline correction in women and sideburn grafts.

According to Unger, hairless lesions secondary to traumatic events (e.g., burns, radiation, prior surgeries, and traction injuries) that can cause permanent scarring in a hair-bearing region are specifically called “stable cicatricial alopecia (SCA)” [1]. Due to the general popularity of facial cosmetic surgeries, such as forehead lifts, forehead implants, and fat grafts, and the increasing numbers of complications involving scalp scars following these operations, there is an increased demand for the treatment of postsurgical alopecia [2–5].

For the most common nonscarring alopecias, such as androgenetic alopecia or alopecia areata, several effective medical treatments have been developed, such as oral finasteride and topical minoxidil [6–8]. Surgical treatment options also exist, including tissue expansion, flap surgery, and hair transplantation; all of these procedures are widely performed with successful results [9–12].

However, many surgeons have found the surgical treatment of traumatic or postsurgical cicatricial alopecia challenging because of tissue stiffness, possible poor blood circulation, and infection. As a result, the excision and direct closure method is preferred over hair transplantation as the primary surgical modality for correcting small alopecia lesions. However, the excisional method often leaves wide scars that widen further due to secondary tension [13–16].

Surgical treatment for postsurgical scar deformity is rarely performed and has been ignored. However, the treatment of even small hairless areas of postsurgical SCA cannot be ignored because these areas can become sources of psychosocial alienation and dissatisfaction [17, 18]. We introduce an effective surgical method for managing postoperative small SCA areas using follicular unit (FU) hair transplantatio, and present a series of cases with aesthetically pleasing outcomes.

## 2. Materials and Methods

From December 2013 to August 2016, 15 patients with postsurgical SCA were included in the study. All patients had scar-induced hairless lesions on their scalps caused by various operations, including forehead implant insertion (n=2), Endotine (Endotine™ forehead bioabsorbable implant, MicroAire Aesthetics, Charlottesville, VA, USA) lift (n=2), previous hair transplantation donor sites (n=5), forehead reduction (n=1), fat grafting (n=1), nevus excision (n=1), and neurosurgeries (n=3). Follicular units were harvested from the occipital area by the strip excision (n=4) or follicular unit extraction (FUE) technique (n=11) using an electronic punch device (Folligraft®, LeadM Corp., Seoul, Republic of Korea) and placed onto the recipient site at the scalp using a hair implanter (Choi Implanter, LeadM Corp., Seoul, Republic of Korea). The affected scarred area on the scalp was calculated by tracing the lesion on millimeter graded transparent paper. Because the desired FU density of the recipient site was approximately 35 units/cm^2^, we could determine the approximate number of FUs to harvest by multiplying the calculated recipient area by the desired density (35 FU/cm^2^).

No additional procedures were performed after the transplantation operations, and all the patients completed their treatment in only one procedural session. Oral antibiotics were administered for 3 postoperative days, and no occlusive dressing was needed except immediate compression of the recipient site and donor site for up to 30 minutes. The patients were able to wash their hair 24 hours after the procedure.

The patients were scheduled to visit the clinic 12 months after the operations so that the graft survival rate could be determined. The Patient and Observer Scar Assessment Scale (POSAS), a promising scar evaluation tool involving both the observer's and the patient's perspectives, was applied for each patient to objectively assess satisfaction. All procedures in the present study were performed in accordance with the Declaration of Helsinki, and approval was granted by the Institutional Review Board of the Seoul National University Boramae Medical Center (IRB No. 10-2018-6).

### 2.1. Surgical Procedure

First, the donor and recipient sites were anesthetized by regional block involving the supraorbital, supratrochlear, and occipital nerves with a mixture of 2% lidocaine and 1:1000 epinephrine. Following nerve blockage, Abbasi solution comprising 100 ml of 0.9% normal saline solution, 5.0 ml of 2% lidocaine, 1 ml of 1:1,000 epinephrine, and 1.0 ml triamcinolone 40 mg/ml was infiltrated throughout the donor and recipient sites [19].

In cases of more than 500 FUs, we performed occipital scalp strip excision (2 to 3 cm wide and 13 to 20 cm long) and extracted the FUs for transplantation. If a small number of less than 500 FUs were needed, we extracted individual FUs using an electronic circular punch with a blunt tip and a diameter of 0.8 mm. The instrument's blunt tip reduced damage to the follicles, and the small holes created by the punch healed within a few days by secondary healing. The extracted FUs were handled gently and kept moist in 0.9% normal saline as the holding solution until they were transplanted. The FUs were sorted by the number of hairs and their thickness such that they could be selectively grafted to appropriate areas of the scalp. The FU grafts were transplanted using a manual implanter with an average density of 35 units/cm^2^ [20–22].

### 2.2. Patient and Observer Scar Assessment Scale (POSAS)

The POSAS was used as an objective assessment tool to analyze satisfaction regarding scarring. It consists of two numeric scales: the Observer Scar Assessment Scale (OSAS) and the Patient Scar Assessment Scale (PSAS). The OSAS includes six domains (vascularity, pigmentation, thickness, relief, pliability, and surface area), each of which is graded numerically on a 10-point scale ranging from 1 (normal skin) to 10 (the worst scar result). The PSAS includes pain, pruritus, color, thickness, surface roughness, and pliability, which are also graded using the 10-point scale. The combination of the patient and observer parts of the scale allows a more complete evaluation.

We used Wilcoxon's signed rank test to compare the preoperative and postoperative POSAS scores, and p values less than 0.05 were considered statistically significant. Statistical results were calculated using IBM SPSS software, version 23.0 (IBM Corp., Armonk, NY, USA).

## 3. Results

In total, 15 patients, including 10 males and 5 females with a mean age of 28.53 years (range 16 to 42 years), were reevaluated 12 months after one procedural session (Table 1). The affected bald area was 16.71 cm^2^ (range 1.20 to 130.00 cm^2^). An average of 584.93 FUs (range 42 to 4,550 FUs) were transplanted with an aesthetically acceptable scar-camouflaging effect, and the mean survival rate was 80.67% (range 70 to 90%). All 15 patients underwent only one procedure session, and the duration of surgery was an average of 1.80 hours (range 0.5 to 5.5 hours). The donor area showed negligible scarring in all cases. No significant complications, such as necrosis, infection, hematoma, or numbness, were observed among the 15 patients.

As shown in Table 2, remarkable improvement was observed after the procedure in terms of patient and observer satisfaction. The PSAS decreased from 13.47 (range 9 to 20) to 6.60 (range 4 to 10), and the OSAS decreased from 11.00 (range 7 to 18) to 5.00 (range 3 to 8) after the procedure. The total postoperative POSAS showed a significant decrease, with a preoperative score of 24.47 (range 16 to 38) and a postoperative score of 11.60 (range 7 to 18), according to Wilcoxon's signed rank test (p=0.001).

### 3.1. Case 1

**Figure 1:**
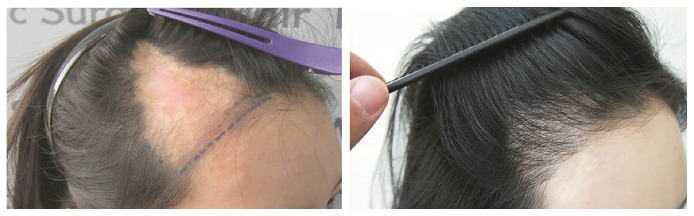
A 19-year-old female patient, who had skin necrosis after undergoing a fat graft to her forehead 1 year previously, had an approximately 130 cm^2^ postsurgical triangular hairless lesion on the right frontotemporal area* (Left)*. In total, 4,550 FUs were extracted and transplanted by the scalp strip excision method, and the graft survival rate was 75% at the 12-month follow-up visit* (Right)*.

A 19-year-old female patient had undergone surgical fat grafting to the forehead with postsurgical sequela of a necrotic hairless lesion, approximately 130 cm^2^ in area, with a triangular shape, that had remained for longer than a year in the right frontotemporal region of her scalp (Figure 1). In total, 4,550 FUs were harvested from the occipital scalp by the strip excision method and transplanted at a density of approximately 35 FUs/cm^2^. The patient underwent a single surgical session that lasted 5.5 hours. At the twelve-month follow-up, the graft survival rate was 75%. The preoperative POSAS was 20 for the patient scale and 18 for the observer scale; the postoperative scores were 8 and 7 for the patient and observer scales, respectively.

### 3.2. Case 2

**Figure 2:**
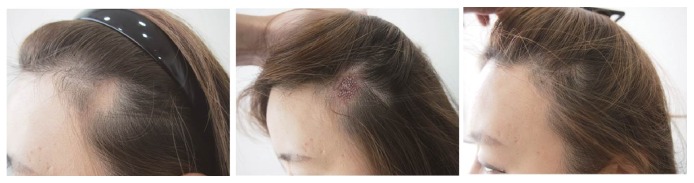
A 42-year-old female patient had a 2 x 3 cm^2^ postsurgical round hairless scar on her left frontotemporal scalp due to skin necrosis after undergoing an Endotine forehead lift two years previously* (Left)*. She underwent hair transplantation of 210 FUs by the FUE method* (Middle)*. After 12 months, the survival rate was 85%* (Right)*.

A 42-year-old female patient had a forehead lift using Endotine fixation (Endotine™ forehead bioabsorbable implant, MicroAire Aesthetics, Charlottesville, VA, USA) two years previously, which led to skin necrosis on her left frontotemporal scalp (Figure 2). The affected area was round and 2 x 3 cm^2^ in area, and 210 FUs harvested from the occipital scalp by the FUE method were transplanted into the lesion at a density of 35 FUs/cm^2^. The operation lasted 1.3 hours, and only a single session was required. The graft survival at the 12-month follow-up was 80%. The preoperative POSAS of the patient scale was 12 and that of the observer scale was 9; the postoperative POSAS was of the patient scale was 5 and that of the observer scale was 3.

### 3.3. Case 3

**Figure 3:**
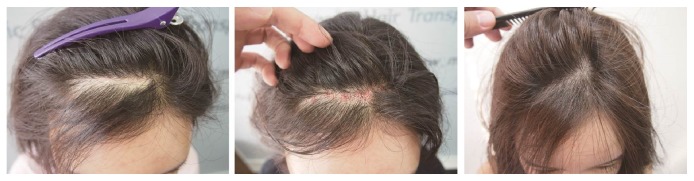
A 33-year-old female patient, who had undergone forehead implant insertion three years previously, had a 7 x 1 cm^2^ postsurgical linear hairless scar on the frontal scalp* (Left)*. A total of 245 FUs were transplanted by the FUE method* (Middle)*, and at her 12-month follow-up visit, the graft survival rate was approximately 85%* (Right)*.

A 33-year-old female had an approximately 7 x 1 cm^2^ lesion consisting of a postsurgical linear scar on her frontal scalp due to forehead implant insertion three years earlier (Figure 3). In total, 245 FUs were harvested from the occipital scalp using the FUE method and transplanted into the lesion. The operation time was 1.5 hours, and the transplantation was performed at a density of 35 FUs/cm^2^. She underwent only one procedural session and achieved an 85% survival rate at the 12-month follow-up. The preoperative POSAS scores were 14 for the patient scale and 13 for the observer scale; the postoperative POSAS scores were 6 for the patient scale and 4 for the observer scale.

### 3.4. Case 4

**Figure 4:**
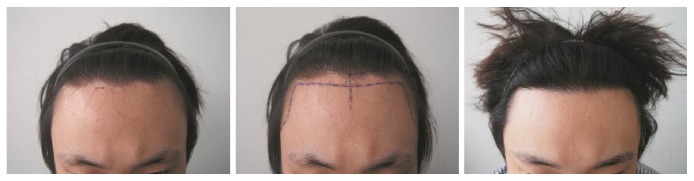
A 28-year-old male patient had complications from an incision line scar near his hair line involving a 20 x 1 cm^2^ area (black arrow) after undergoing forehead reduction surgery one year previously* (Left)*. In total, 700 FUs harvested from the occipital scalp were transplanted onto the postsurgical alopecia lesion* (Middle)*. The final graft survival rate at the 12-month follow-up was 80%* (Right)*.

A 28-year-old male patient with a wide forehead had undergone a forehead reduction surgery one year previously, which resulted in a postsurgical linear hairless scar approximately 20 x 1 cm^2^ in area near his hairline (Figure 4). A total of 700 FUs were harvested from the occipital scalp by the FUE method and transplanted into the lesion at a density of 35 FUs/cm^2^. The operation lasted 2.5 hours, and a single session was required. At his 12-month follow-up visit, the graft survival rate was approximately 80%, and the POSAS had decreased from 10 on the patient scale and 8 on the observer scale preoperatively to 6 and 4, respectively, at the postoperative evaluation. Instead of performing forehead reduction surgery, which results in an incision line scar as a secondary complication, hair transplantation can be used as a primary treatment for camouflaging a wide forehead.

## 4. Discussion

Cicatricial alopecia encompasses a diverse spectrum of disorders characterized by permanent destruction of hair follicles and replacement by fibrous tracts. Unger et al. defined two categories of cicatricial alopecia: stable and unstable. Unstable cicatricial alopecia is the consequence of progressive dermatologic disorders that can recur intermittently over time, such as lichen planopilaris and discoid lupus erythematosus. In contrast, SCAs occur secondary to traumatic events, such as prior surgeries, burns, radiation, and traction injuries, that can cause permanent scarring in hair-bearing regions [1, 23, 24].

Our study focused on surgically induced SCA. The traditional surgical treatment methods for such lesions include excision, local flap, and tissue expansion, which can result in additional scarring, an unfavorable hair growth direction, and vascular network compromises [1, 5]. Some reports have described successful cases of camouflaging postsurgical scar alopecia with hair transplantation [14, 25].

Since the first hair transplantation was introduced by a Japanese dermatologist, Sasagawa, in 1930, hair transplantation techniques have developed further [26]. In 2002, the FUE technique was introduced by Rassman and Bernstein as an alternative method for harvesting scalp strips from the occipital area with the advantage of no need for sutures and no linear scar. However, this method can still cause problems, such as a wider donor area, spot scars, and transection of follicles if performed by an unskilled surgeon. Therefore, care should be taken to reduce these side effects.

The concept of implanting hair follicles has not been widely accepted as a primary treatment for postsurgically occurring alopecia with the presumption that the graft survival rate would be low due to limited vascularity, tissue stiffness, skin thinning, and possible infection at the recipient site [27, 28]. Conversely, some previous studies have shown that, because of the small size and low metabolic requirement of FUs, hair grafts can grow well in scar tissue [13, 25, 29]. According to Shao, hair transplantation in 37 cicatricial alopecia patients with traumatic scars resulted in a 78% survival rate at an average of 13 months of follow-up [14]. Additionally, Jung showed that, among 18 patients who underwent hair follicle transplantation onto posttraumatic scar tissue, 15 showed more than 75% graft survival at the 6-month follow-up visit [5].

In our clinical study, the survival rate of FU transplants ranged from 70 to 90%, with an average of 80.67%. In our clinical study, the survival rate of the FU transplantations ranged from 70 to 90% with an average of 80.67%. This rate is lower than the average survival rate of 90% in normal scalps from our previous experience and previous studies [25, 30–32]. Furthermore, in some cases, the survival rates remained as low as 70%, which is much lower than the average in normal scalps. However, the average survival of 80% is still meaningful because this rate is much higher than our expectation, and the postoperative outcomes were aesthetically pleasing to both the patients and observers.

The overall patient satisfaction was also very high not only because the transplantation results were anesthetically pleasing but also because no potential existed for the development of flap ischemia or exposure of tissue expanders, which could have occurred with traditional surgical methods, such as scar excision, tissue expansion, and local flaps. Additionally, there was no need for general anesthesia or admission care because the procedure lasted only 1.80 hours on average and did not require any specific postoperative care. All patients were treated at an out-patient clinic with minimal recovery time. There was no need for any dressing of small grafts. Only immediate compression for less than 30 minutes was performed at the recipient and donor sites. The patients were able to wash their hair one day after the procedure and to continue with their daily lives.

The approximate density of hair transplantation in this study was 35 FUs/cm^2^, which was determined by the practitioner's previous experiences. Although debates are ongoing, it is widely accepted that the transplantation of more than 35 FUs/cm^2^ onto normal tissue can induce poor graft growth and even skin necrosis depending on the vascularity of the recipient site [20–22]. In scar tissues, the vascular supply that supports the newly transplanted grafts must be considered carefully in the preoperative evaluation. Additionally, in this study, the transplantation procedure was performed at least 6 months after the operations that induced the postsurgical scar lesions in the original locations. Hair transplantation at a density of 35 FUs/cm^2^ more than 6 months after the original operation resulted in a relatively successful survival rate with no complications, such as skin necrosis, infection, hematoma, and numbness. It is presumable that, after 6 months of recovery, scar tissue on the scalp can supply blood for FU transplantation at an almost similar density as normal scalp tissue.

When grafting onto very thin attenuated skin, grafts should be placed at an acute angle because the tissue bed is very shallow. The use of tumescence before hair transplantation could facilitate the proper placement and optimal growth of grafts in compromised tissues [25]. In this study, Abbasi solution was used to achieve temporary thickness to facilitate easier dissection and implantation, better hemostasis, and adequate anesthesia. Moreover, triamcinolone was added to help minimize postoperative edema and pain.

This study was conducted with only 15 patients over a period of less than 3 years. Further studies should be conducted in the future with additional patients with various affected areas and different types of postsurgical cicatricial alopecia and over longer periods. Specifically, the difference in the histological background of the underlying infrastructure found in the dermis and along the hair follicular unit between the normal scalps and postsurgical scalps with scars should be identified to explain the different hair graft survival rates. Additionally, the average graft survival rate in this study was 80.67%, which was higher than that previously expected for hair transplantation in cicatricial alopecia but still lower than the average survival rate for normal tissues. Various approaches, such as preoperative fat grafts and adipose stem cell grafts, could be combined in advance with hair transplantation to increase the quality and pliability of scar tissue and ultimately increase the graft survival rate [33–35].

## 5. Conclusions

Treatment of even small hairless lesions is important because it can affect the patient's psychosocial status. However, hair transplantation for postsurgical SCA is considered challenging because of tissue stiffness and poor blood circulation. FU hair transplantation could be an effective method with several advantages, including relative safety, a high transplantation survival rate, and satisfactory postoperative results, even after a single session.

## Figures and Tables

**Table 1:** Patient Characteristics.

*Case*	*Sex*	*Age (years)*	*Etiology*	*Location*	*Shape*	*Size (cm* ^*2*^)	*Transplanted graft (FU)*	*Survival rate at 12 MO *(%)	*Operation time (hours)*
1	F	19	Fat graft	Rt. frontotemporal	Triangular	130	4,550	75	5.5
2	F	42	Endotine lift	Lt. frontotemporal	Round	6	210	85	1.3
3	F	33	Forehead implant insertion	Frontal	Linear	7	245	85	1.5
4	M	28	Forehead reduction	Frontal	Linear	20	700	80	2.5
5	M	18	Neurosurgery	Bilateral frontotemporal	Linear	10	350	75	1.5
6	F	41	Forehead implant insertion	Frontal	Linear	1.2	42	90	0.5
7	M	16	Neurosurgery	Occipital	Geographic	18	630	75	2.5
8	M	28	Previous hair transplantation scalp strip harvesting site	Occipital	Linear	7.5	262	80	1.5
9	F	40	Endotine lift	Bilateral frontotemporal	Geographic	10	350	85	1.8
10	M	25	Previous hair transplantation scalp strip harvesting site	Occipital	Linear	6	210	80	1.5
11	M	18	Nevus excision	Lt. temporal	Linear	2	70	75	0.5
12	M	28	Neurosurgery	Rt. occipital	Round	15	525	70	2.5
13	M	32	Previous hair transplantation scalp strip harvesting site	Occipital	Linear	6	210	90	1.5
14	M	27	Previous hair transplantation scalp strip harvesting site	Occipital	Linear	6	210	80	1.2
15	M	33	Previous hair transplantation scalp strip harvesting site	Occipital	Linear	6	210	85	1.2
Average ± SD		28.53 ± 8.48				16.71 ± 31.79	584.93 ± 1112.71	80.67 ± 5.94	1.80 ± 1.19

FU, follicular unit; MO, months; SD, standard deviation.

**Table 2:** Preoperative and Postoperative POSAS Assessment.

*Etiology*	*Survival rate at 12 MO *(%)	*Preoperative. POSAS*			*Postoperative POSAS*		
		*Patient*	*Observer*	*Total*	*Patient*	*Observer*	*Total*
Forehead implant insertion (n=2)	87.50	11.50	10.00	21.50	5.00	3.50	8.50
Endotine lift (n=2)	85.00	12.50	9.50	22.00	4.50	3.50	8.00
Previous hair transplantation scalp strip harvesting site (n=5)	83.00	11.60	8.80	20.40	6.40	4.80	11.20
Forehead reduction (n=1)	80.00	10.00	8.00	18.00	6.00	4.00	10.00
Fat graft (n=1)	75.00	20.00	18.00	38.00	8.00	7.00	15.00
Nevus excision (n=1)	75.00	16.00	14.00	30.00	8.00	7.00	15.00
Neurosurgery (n=3)	73.33	16.67	14.00	30.67	8.67	6.33	15.00
Average ± SD	80.67 ± 5.94	13.47 ± 3.48	11.00 ± 3.46	24.47 ± 6.61	6.60 ± 1.92	5.00 ± 1.77	11.60 ± 3.54

POSAS, The Patient and Observer Scar Assessment Scale; MO, months; SD, standard deviation.

## Data Availability

The data used to support the findings of this study are available from the corresponding author upon request.
